# Are men dominant? Evidence of differences between physical activity and quality of life among older adults in China

**DOI:** 10.3389/fpubh.2023.1210374

**Published:** 2023-09-29

**Authors:** Mo Chen, Shanping Chen, Yuyan Wu, Di Song, Lijun Xie, Yao Shang, Zhiyi Chen

**Affiliations:** Department of Physical Education, Xi'an Jiaotong University, Xi'an, China

**Keywords:** aging population, older men, healthy, exercise, SF-36, healthy aging

## Abstract

At present, the aging population is one of China's basic national concerns, and physical exercise offers endless potential to cope with it. However, the life expectancy of men in China is generally lower than that of women, and the health status of older men is more worrying. Could it be that differences in physical exercise cause the difference in life expectancy between older men and women? This study analyzes the exercise regimen of older men and women and its influence on their quality of life. Approximately 200 respondents aged over 60 were investigated using the SF-36 and exercise questionnaires. Our findings revealed the following: (1) The scores of seven dimensions of life quality of older men were significantly lower than those of older women (*p* < 0.001), but there was no significant difference only in physiological function (*p* > 0.05); (2) The exercise frequency and persistence of older men were significantly lower than those of older women (*p* < 0.001), but there was no significant difference in exercise time (*p* > 0.05); and (3) All eight indices of quality of life of older men were positively correlated with the four indices of exercise (0.250 < *R* < 0.597). Our study offered the following conclusions: (1) The health of older men who lack physical exercise is poor. From the perspective of healthy aging, older men are a vulnerable group that deserves more attention. (2) Within an appropriate range, the more older men participate in physical activity programs, the more conducive they are to improved health. (3) This study focuses on promoting physical exercise for older adults and suggests organizing them to participate in sports activities as an important measure to promote healthy aging in China.

## 1. Introduction

China has the largest aging population in the world. Along with the increasing aging, the associated health problems place an extremely heavy burden on the whole country (1). The results of the seventh national census show that “China's population aged 60 and above exceeds 260 million, accounting for 18.70% of the population, and aging is intensifying” (2). In this context, how to achieve healthy aging in an economical and effective manner is a major dilemma that China faces. Cognizant of the challenges posed by population aging, the Chinese government has given its full consideration and attention to it. It has incorporated development strategies and countermeasures that address aging when formulating the National Economic and Social Development Plan; it has prioritized the wellbeing of the older population to ensure they enjoy a healthy old age (3). In the “Health China 2030 Plan,” “strengthening health services for key populations” explicitly mentions the need to “promote healthy aging.” The plan proposes to “strengthen the integration of physical medicine and non-medical health interventions” and “promote physical activities for... the older... and other key populations” (4). Therefore, the development of sports and physical activities for the older adult population is an important way to promote healthy aging in China. Furthermore, it is a topic worthy of in-depth study that can help promote national fitness and aid China in becoming a “strong sports nation” (5).

With a growing aging population and national attention on it, the health status of this demographic has attracted extensive academic attention and research. Numerous studies have shown that regular physical activity has many health benefits for older adults, such as improving physical and mental health (6), preventing diseases (7), and reducing the risk of chronic diseases (8, 9). According to experts, middle-aged and older adults who participate in at least 150 min of moderate-to-vigorous physical activity per week can reduce mortality by 31%, the rate of cognitive decline by about 1/3, the risk of stroke by 15%, and the relative risk of activity limitation by 50% (7).

Studies on population longevity have shown that women live significantly longer than men and there are significant differences in health parameters between older men and women (10). In Chinese tradition, women are considered weak, and men are considered strong. Therefore, it is customary to focus more on women, which has resulted in not enough attention being paid to men, their physical fitness, and their health, leading to their neglect (11). In the existing literature, there are many studies focusing on the health and physical education of older adults. However, from a gender perspective, researchers have neglected promoting the health and physical exercise of older men. In particular, literature combining physical exercise and health for older men is scarce. In order to achieve comprehensive healthy aging, the health of the entire aging population must be studied and promoted. Therefore, this study aims to analyze the physical exercise regimen of older men and the effect it has on the quality of their life from the perspective of gender differences in order to provide a reference for promoting the quality of life of older men and achieving healthy aging in China.

## 2. Materials and methods

### 2.1. Participants

The target population of this study was older adults. In order to analyze from a gender perspective, the survey population was recruited using male and female pairs to select 200 older adults aged over 60 years (paired older people were drawn from the same community). In order to control the influence of irrelevant variables such as community environment, the questionnaire survey selected several communities in Xi'an with minimal differences between them in living conditions for questionnaire distribution. Inclusion criteria included being able to move around the community without barriers, agreeing to participate in the questionnaire, having some cognitive ability, and being able to answer the investigator's questions. Exclusion criteria included the presence of cognitive impairment, unable to effectively answer the investigator's questions, and the presence of physical disability or apparent disease. The basic information of the survey respondents is shown in Table 1.

**Table 1:** Basic information of survey respondents (*n* = 200).

Basic information^*^	**Category**	**Number of people**	**Proportion**
Gender	Male	100	50%
	Female	100	50%
Age	60–64 years old	66	33%
	65–69 years old	38	19%
	70–74 years old	39	19.5%
	75–79 years old	32	16%
	80 years old and above	25	12.5%
Academic qualifications	Primary school	16	8%
	Junior high school	49	24.5%
	High school	48	24%
	Secondary school	31	15.5%
	College	34	17%
	Undergraduate	19	9.5%
	Graduate and above	3	1.5%
Working situation	On-the-job	16	8%
	Retired	184	92%

^*^Data information: Xi'an City, Shaanxi Province, 2017.

### 2.2. Measurement tools

The Quality of Life Assessment uses the SF-36 Quality of Life Self-Measurement Scale issued by the World Health Organization. There are 36 questions with eight dimensions, including Physical Functioning (PF), Role-Physical (RP), Bodily Pain (BP), General Health (GH), Vitality (VT), Social Functioning (SF), Role-Emotional (RE), and Mental Health (MH). While PF measures whether health conditions interfere with normal physical activities, RP measures functional limitations due to physical health problems, and BP measures pain levels and the impact of pain on daily activities. On the other hand, GH is a subjective measure of the respondent's evaluation of his or her own health status and future health trends. VT measures the individual's subjective feelings about their energy and fatigue levels, and SF measures the individual's social function in terms of the impact of physical and psychological problems on the quantity and quality of the individual's social activities and is used to evaluate the effect of health on social activities. RE measures the functional limitations due to emotional problems, while MH measures whether the respondent is mentally healthy and assesses whether the individual is mentally tense, depressed, or restless and whether the individual is happy and calm. The first four dimensions together reflect the physical health of the respondent, and the last four dimensions observe the person's mental health. The consistency reliability coefficient of the scale was 0.72–0.88, and the 2-week retest reliability was 0.66–0.94 (12). The scoring method of the SF-36 was adapted from the method introduced by Chonghua Wan in Introduction to Commonly Used Quality of Life Measurement Scales (13).

The physical exercise behavior assessment uses four indicators, including exercise items, exercise time, exercise frequency, and exercise behavior stage. The exercise item test question included questions such as: “The items you often participate in physical exercise (multiple choice): (1) running, (2) basketball, (3) soccer, (4) table tennis, (5) badminton, (6) swimming, (7) aerobics, (8) dance, (9) martial arts, (10) taijiquan, (11) qigong, (12) yoga, (13) walking, (14) gateball.” Exercise time refers to the time spent on physical exercise each time, and it included test question such as: “Usually, the time you take part in physical exercise each time: (1) 15 min, (2) 15 to 30 min, (3) 30 min to 1 h, (4) 1 to 1.5 h, (5) more than 1.5 h.” Exercise frequency refers to the number of times the respondents participated in physical exercise each week, and it had test questions such as: “Usually, you participate in physical exercise each week: (1) <once, (2) once, (3) twice, (4) three times, and (5) four times and more.” The exercise behavior stage measurement question included questions such as: “Regular physical exercise is defined as planned physical activity to promote health, such as walking, jogging, ball games, and other activities”. Effective exercise should be adhered to more than three times a week, and each time, more than 20 min can make you sweat. According to this definition, do you perform regular exercise? 1. Yes, I have been adhering to such physical exercise for more than 6 months. 2. Yes, I have not been adhering to such physical exercise for more than 6 months. 3. No, I participate in some physical exercise, but not on a regular basis. 4. No, but I intend to start physical exercise. 5. No, and I have no intention to start physical exercise” (14).

### 2.3. Data analysis

Data analyses were performed using SPSS for Windows version 26.0. Statistical methods mainly used independent sample *t*-test and Pearson correlation analysis. Specifically, first, an independent sample *t*-test was used to compare the mean values of the 8 dimensions of quality of life scores between older men and women and to test whether there was a significant difference in the quality of life of older adults by gender. Second, an independent sample *t*-test was used to analyze the comparison of the mean values of the number of sports, exercise frequency, exercise time, and exercise persistence between older men and women and to test whether there was a significant difference in the physical activity behavior of the study participants by gender. Finally, Pearson correlation analysis was used to analyze the correlation between the quality of life and physical activity behavior of older men. The analysis was aimed to determine the significant effects of different numbers of sports programs, exercise frequency, exercise time, and exercise persistence on the quality of life scores of older men assessed across the eight dimensions.

## 3. Results

### 3.1. Comparative analysis of the quality of life of older men and women

The Quality of Life Measurement Scale assesses a number of aspects of physical, mental, and social health. Table 2 provides the results of the statistical analysis of the independent samples *t*-test for the scores of the eight dimensions of quality of life in older men and women. From the statistical results, it is apparent that older men scored significantly lower (*p* < 0.001) than older women in 7 dimensions of quality of life, including Role-Physical (41.75), Bodily Pain (51.50), General Health (56.65), Vitality (64.20), Social Functioning (77.50), Role-Emotional (62.66), and Mental Health (65.36), with a non-significant difference (*p* > 0.05) only in Physical Functioning (75.70). This indicates that the quality of life of older men is poor, and as a health-disadvantaged group, they should be the focus of attention from the perspective of healthy aging.

**Table 2:** Independent sample *t*-test for quality of life in older men and women.

	**Older men**		**Older women**		***t***	**sig**
	**Mean**	**St.d**	**Mean**	**St.d**		
Physical Functioning (PF)	75.700	17.437	77.750	15.397	−0.881	0.379
Role-Physical (RP)	41.750	35.543	72.250	38.088	−5.855	0.000
Bodily Pain (BP)	51.500	13.734	64.500	13.734	−6.693	0.000
General Health (GH)	56.650	11.504	62.750	7.797	−4.389	0.000
Vitality (VT)	64.200	12.221	72.900	11.938	−5.092	0.000
Social Functioning (SF)	77.500	21.022	93.500	18.333	−5.736	0.000
Role-Emotional (RE)	62.660	37.084	83.992	30.141	−4.464	0.000
Mental Health (MH)	65.360	11.317	72.800	11.456	−4.620	0.000

### 3.2. Comparative analysis of physical activity behavior of older men and women

The physical activity behavior assessment includes the following four indicators: exercise items, exercise frequency, exercise time, and exercise behavior stage. The exercise items include the names of the sports items, which are used to calculate the number of sports items the subjects participate in; the exercise frequency is used to analyze the number of times the subjects participate in physical exercise in a week; the exercise time is used to analyze the length of time the subjects participate in each physical exercise; and the exercise behavior stage is used to analyze whether the subjects engage in regular, healthy and planned physical exercise.

Table 3 gives the results of the statistical analysis of the independent sample *t*-test for the physical activity behavior of older men and women. From the statistical results, it is evident that older men scored significantly lower than older women in the number of participating sports (1.40), exercise frequency (2.92), and exercise adherence (3.15) (*p* < 0.001). The difference in exercise time (2.94) was not significant (*p* > 0.05). It indicates that the current physical exercise behavior of older men is less than that of older women. In terms of the mean value, the number of sports items that older men frequently participate in is 1.4; the number of times they participate in physical exercise per week is mainly 1 to 2 times; the duration of each participation in physical exercise is mainly within 30 min; and the stage of exercise behavior is mainly in the preparation stage, which is expressed as “participating in some physical exercise, but not regularly.” This is consistent with our observation that older men participated less in physical activity compared to women.

**Table 3:** Independent sample *t*-test for physical exercise behavior of older men and women.

	**Older men**		**Older women**		***t***	**sig**
	**Mean**	**St.d**	**Mean**	**St.d**		
Sports items	1.400	0.651	1.900	1.068	−3.996	0.000
Exercise frequency	2.920	1.022	3.960	0.898	−7.645	0.000
Exercise time	2.940	0.930	3.080	0.837	−1.119	0.265
Exercise adherence	3.150	1.077	4.220	0.970	−7.384	0.000

### 3.3. Correlation analysis of quality of life and physical activity in older men

In order to analyze whether there is a relationship between the poor quality of life of older men and their lack of physical exercise, the correlation between their quality of life and physical exercise behavior data was conducted, and the results of the analysis are shown in Table 4. From the data in Table 4, it can be seen that all eight indicators of quality of life of older men have a significant positive correlation with four indicators of exercise behavior (0.250 <*R* < 0.597). The correlation coefficients of Physical Functioning, General Health, Vitality, Social Functioning, Mental Health, and exercise behavior reached 0.001. This indicates that, within the scope of their ability, the more older men participate in physical exercise programs, the more frequently they exercise, the longer the duration of each physical exercise, and the longer they persist in exercising, the better their quality of life would be.

**Table 4:** Correlation analysis of quality of life and physical activity in older men.

	**PF**	**RP**	**BP**	**GH**	**VT**	**SF**	**RE**	**MH**
Sports items	0.429^***^	0.253^*^	0.203^*^	0.369^***^	0.377^***^	0.369^***^	0.262^**^	0.413^***^
Exercise frequency	0.502^***^	0.253^*^	0.333^**^	0.518^***^	0.593^***^	0.474^***^	0.231^*^	0.471^***^
Exercise time	0.597^***^	0.321^**^	0.284^**^	0.278^**^	0.524^***^	0.492^***^	0.422^***^	0.492^***^
Exercise adherence	0.395^***^	0.250^*^	0.333^**^	0.465^***^	0.585^***^	0.391^***^	0.226^*^	0.487^***^

^*^p <0.05; ^**^p <0.01; ^***^p <0.001.

## 4. Discussion

### 4.1. Quality of life for older men requires focused attention

With an increasing aging population, the health of older adults in China has been of much concern and research. However, the influence of traditional Chinese concepts such as “strong male and weak female” has often resulted in the neglect of older men. From the results presented in Table 1, it can be seen that the scores of older women in the eight dimensions of quality of life are higher than for men, and there are significant differences between older men and women in the remaining seven dimensions except for physiological function (PF), which indicates that the current health condition of older men is not optimistic. The ability of older men to cope with adverse events decreases with age. For example, adverse events such as retirement, widowhood, and family discord can have significant effects on older men's physical functioning (RP), social functioning (SF), somatic pain (BP), general health self-assessment (GH), and emotional functioning (RE) (15). It has also been suggested that anxiety is more severe among older men without a spouse (16). After the transition from middle age to old age, health concerns are often not as important as those of women of the same age, and there are relatively few exercise prescriptions for older men (17). Currently, more and more older men are suffering from cardiovascular diseases, which are caused by bad habits such as socializing, drinking, and staying up all night during middle age, which affect their energy (VT) and body pain (BP) and often affect their work and daily life. As older men shoulder important family responsibilities, they deserve more attention (18). In recent years, the country has actively implemented a national strategy to cope with population aging and integrated the concept of positive and healthy aging into the whole process of economic and social development (19). Meanwhile, in the context of general health, the poor quality of life of older men is a serious problem, and as a vulnerable group, they deserve the attention of society.

### 4.2. Lack of physical activity in older men predisposes them to poor quality of life

The reasons for the poor health of older men are many. On the one hand, it is related to their age, where with the progression of age, their physical condition declines, and the function of the body organs gradually deteriorates, resulting in poor health. On the other hand, men tend to assume more responsibility in society and family, balancing work and family commitments. However, they are also prone to various chronic diseases caused by their overwork and exertion. For example, stress can lead to heart disease and stroke in older adults reducing the quality of life (20). Another very important reason is the lack of physical activity (21). Table 2 shows that the number of physical exercise programs for older men (1.40) is lower than the number of physical exercise programs for older women (1.90), which is consistent with the existing research literature. In other words, the choice of physical exercise programs for older women is richer, while the choice of physical exercise programs for older men is relatively limited. The study by Li and Guan (22) found that older men's lack of interest in sports programs and their numerous responsibilities contribute to their lack of exercise.

Table 2 shows that the frequency of physical exercise in older men (2.92) is lower than that of older women (3.08). Compared to women, older men are generally less physically active in their daily lives. In recent years, older women were found to have more free time after retirement than before, which allowed them to participate in sports more (23). In contrast, older men in the same age category are less involved in physical activity compared to women. Some studies have shown that one of the important reasons for the low frequency of physical activity among older men is related to the lack of scientific knowledge of exercise (24). In addition, factors such as bad weather, congested traffic, and lack of guidance from community sports personnel are also important factors that affect the frequency of physical activity among older adults (25). Table 2 also reveals that older men's exercise persistence (3.15) is significantly lower than that of older women (4.22). Some studies suggest that the degree of improvement of the public sports service system affects older men's motivation to participate in physical exercise (26), which in turn affects their exercise persistence. For example, most of the sports provision for older men is in squares, parks, and fitness venues in the community. However, the supply of public sports venues, fitness centers, and clubs for older men is not perfect, and the lack of or damage to sports venues and sports equipment facilities is one of the important reasons for their lower exercise adherence. This series of problems can affect older men's behavior of engaging in physical exercise, which in turn affects their quality of life. Inadequate physical activity in older men leads to poor quality of life, and the role of physical activity on physical health should not be ignored.

### 4.3. Strengthening sports services for older men to improve their quality of life

Physical education is an important means of promoting human development and health. Not only does physical exercise improve one's physical health and physical activity, but physical exercise can also relax the brain, relieve psychological stress, eliminate tension, develop a positive outlook on life, and thus improve mental health. More studies have confirmed that physical activity can effectively promote the quality of life of individuals (27, 28). For example, Wang et al. (29) analyzed the factors influencing the quality of life of secondary school teachers in Lanzhou City and found that the scores in each domain tended to increase as the duration of exercise increased. O'Brien et al. (30) undertook a 1-year quality of life and health status survey of COVID-19 inpatients and also argued that physical activity at suboptimal levels facilitated the recovery of some objective physical functions. Min-Hsiung Chen found that regular physical activity increased bone mineral density in older men, thereby reducing the incidence of osteoporosis (31). Li (17) proposed that active scientific exercise in older adults can effectively improve the function of various systems, achieve physical and mental health, and improve the quality of life. Similarly, Li (32) proposed that physical exercise as a form of social activity effectively increases the social communication opportunities of older adults and has a positive impact on them. Liu (33) proposed in the study of the effect of physical exercise on the quality of life of older adults that the effect of physical exercise on the physiological health of older adults was significantly correlated. Furthermore, older adults with a high amount of physical exercise had fewer chronic diseases, and the correlation analysis of our study on the quality of life and physical exercise (Table 3) further confirmed that physical exercise also had a significant contribution to the quality of life of older men.

There are many studies suggesting that older adults should increase the frequency and duration of physical activity (34, 35). To promote healthy aging, enhancing the time of physical exercise, frequency of physical exercise, and duration of physical exercise among older men should be the main goal. Furthermore, strengthening the sports support services for older men and organizing the participation of older men in sports activities should be implemented as an important measure. The main measures should be reflected in three major aspects, including promoting the scientific nature of exercise, improving the richness of exercise programs, and improving venue facilities.

First, using modern information technology, efforts should be made to publicize, promote, and raise awareness of the scientific methods and means of fitness and exercise for older men. At the same time, older men should be given enough sports guidance to make their physical exercise scientific and standardized, which will also reduce the occurrence of injury and accidents. Second, according to Zhou et al. (36) and research on the current state of physical exercise for older adults in Fujian Province, it is recommended to focus on and strengthen the organization and management of physical exercise for older adults through community participation. Meanwhile, communities should actively add diversified physical education courses for the older population to promote the richness of sports programs and improve the quality of life of older adults (37). Local communities should also fully make use of available opportunities in organizing, managing, and promoting sports activities for older men within their jurisdictions and promote the continuous enrichment of sports programs for older men. Third, in the construction of community sports and fitness facilities, certain human, material, and financial resources should be earmarked for sports facilities for older men, and the provision of sports venues and equipment for older men should be increased to create a better environment and conditions for older men to exercise. Strengthening sports services for older men can improve their quality of life. In addition to the above analysis of the quality of life and physical exercise of older men, it can be concluded that an important reason for the poor quality of life of older men is the serious lack of physical activity behavior of older men. This study highlights the importance of scientific and reasonable exercise for older men in promoting healthy aging. To fully maximize the benefits of physical exercise, a collaborative implementation path involving the government, society, and individuals is necessary. By working together, we can truly maximize the potential of physical exercise as a catalyst for healthy aging.

## 5. Conclusion

Our study had the following key observations: (1) The health of older men who lack physical exercise is poor. From the perspective of healthy aging, older men are a vulnerable group that deserves more attention; (2) Within reasonable limits, the more older men participate in physical activity programs, the more conducive they would be to improved health; and (3) This study focused on promoting physical exercise and healthy aging for older adults in China; organizing sports activities for older men is an important measure in promoting healthy aging.

## Data availability statement

The original contributions presented in the study are included in the article/supplementary material, further inquiries can be directed to the corresponding author.

## Author contributions

Formal analysis, writing, data curation, and editing: MC and SC. Original draft preparation: YW and DS. Investigation: MC and LX. Revised the manuscript: MC, SC, YW, DS, LX, YS, and ZC. All authors have read and agreed to the published version of the manuscript.
